# Increasing research on personalized alignment concepts, robotic‐assisted surgery and artificial intelligence in total knee arthroplasty: A bibliometric analysis of the last 10 years in *KSSTA*

**DOI:** 10.1002/jeo2.70798

**Published:** 2026-06-15

**Authors:** Yuma Onoi, Randa Elsheikh, Tomoyuki Matsumoto, Ryosuke Kuroda, Rolf Huegli, Andrej Maria Nowakowski, Michael Tobias Hirschmann

**Affiliations:** ^1^ University Department of Orthopedic Surgery and Traumatology Kantonsspital Baselland Bruderholz Switzerland; ^2^ Department of Clinical Research, Research Group Michael T. Hirschmann, Regenerative Medicine and Biomechanics University of Basel Basel Switzerland; ^3^ Department of Orthopedic Surgery Kobe University Graduate School of Medicine Kobe Hyogo Japan; ^4^ Institute of Radiology and Nuclear Medicine, Kantonsspital Baselland Bruderholz Switzerland

**Keywords:** artificial intelligence, bibliometric analysis, functional alignment, kinematic alignment, robotic‐assisted surgery, total knee arthroplasty

## Abstract

**Purpose:**

To evaluate contemporary trends in total knee arthroplasty (TKA) research published in *Knee Surgery, Sports Traumatology, Arthroscopy* (*KSSTA*) over the last decade, with a focus on alignment concepts and surgical assistance technologies.

**Methods:**

*KSSTA* articles on primary TKA published between 2015 and 2024 were identified through Scopus. After exclusions, 856 articles were analysed. Bibliometric data were extracted and manually classified according to alignment concepts (mechanical [MA], kinematic [KA] or functional [FA]), surgical assistance technologies (manual, navigation, patient‐specific instrumentation [PSI] or robotic‐assisted surgery), study design and the use of patient‐reported outcome measures (PROMs). Analyses and visualizations were performed using R (Bibliometrix) and VOSviewer.

**Results:**

Alignment concepts shifted after 2022; KA increased to 40% (16/40 articles) and FA to 30% (12/40 articles) in 2024, whereas MA declined to 30% (12/40 articles). Manual TKA dominated overall (455/699 articles, 65%), whereas navigation (118 articles, 17%) and PSI (63 articles, 9%) contributed steadily at lower levels. Robotic‐assisted surgery emerged after 2021, overall reaching 63/699 articles (9%) and peaking at 26/104 articles (25%) in 2023. Artificial intelligence (AI)‐based studies appeared after 2020, reaching 6/112 articles (5%) in 2022, showing steady growth. International collaboration rates were higher in Europe and North America (up to ~58% multiple‐country publications [MCP]) than in Asia (<10% MCP). PROMs usage remained broad, with a notable increase in Forgotten Joint Score since 2020.

**Conclusions:**

The *KSSTA* TKA literature (2015–2024) reflects a shift toward personalization (KA/FA) and precision (robotics), alongside the emergence of AI. The convergence of phenotype‐aware planning, precise execution and data‐driven evaluation outlines a coherent path forward. Continued methodological rigour and standardized reporting are essential to translate these trends into durable improvements in TKA outcomes. Future research should prioritize high‐quality comparative studies to clarify the clinical impact of emerging alignment strategies and technological innovations in TKA.

**Level of Evidence:**

Level IV.

**Abbreviations**
AI: artificial intelligence
CPAK: Coronal Plane Alignment of the Knee
FA: functional alignment
FJS: Forgotten Joint Score
KA: kinematic alignment
KOOS: Knee Injury and Osteoarthritis Outcome Score
KSS: Knee Society Score
*KSSTA*
: *Knee Surgery, Sports Traumatology, Arthroscopy*
LoE: levels of evidence
MA: mechanical alignment
MCP: multiple‐country publications
OA: osteoarthritis
OKS: Oxford Knee Score
PROMs: patient‐reported outcome measures
PSI: patient‐specific instrumentation
RCT: randomized controlled trial
TKA: total knee arthroplasty
VAS: Visual Analogue Scale
WOMAC: Western Ontario and McMaster Universities Osteoarthritis Index

## INTRODUCTION

Total knee arthroplasty (TKA) is a well‐established procedure for end‐stage osteoarthritis (OA), yet 10%–20% of patients remain dissatisfied with their outcomes [8]. This persistent dissatisfaction underscores the need for continuous improvements in surgical techniques, implant design, perioperative care and rehabilitation [14, 37]. Although numerous studies have been conducted worldwide, the increasing diversity of research designs and methodologies has made it difficult to identify overarching trends [5, 31]. To address this challenge, bibliometric analysis—which objectively evaluates publication characteristics and emerging research themes—has become an important tool for capturing such trends in orthopaedic research.

Several bibliometric reviews have examined global and regional TKA research and have consistently demonstrated rising output and evolving priorities [28, 29]. A previous *Knee Surgery, Sports Traumatology, Arthroscopy* (*KSSTA*)‐based analysis (2006–2020) [17] reported growing arthroplasty publications, with a shift in focus from navigation toward perioperative management, robotics and personalized surgery. However, that analysis was performed prior to the rapid expansion of concepts, such as kinematic alignment (KA) and functional alignment (FA), and thus did not evaluate their relative representation in the literature or publication trends over time.

Since 2020, alignment strategies have increasingly shifted from traditional mechanical alignment (MA) toward KA and FA [15, 21], while technological assistance has progressed from navigation and patient‐specific instrumentation (PSI) to robotic‐assisted systems [20]. In parallel, knee phenotyping concepts have emerged, including Coronal Plane Alignment of the Knee (CPAK) classification [4, 22]. Additionally, artificial intelligence (AI) has increasingly complemented musculoskeletal research through applications in imaging analysis, phenotype characterization and outcome prediction [19, 32]. These innovations—KA/FA philosophies, robotics and AI—are now widely recognized as key drivers of change in TKA; however, their quantitative representation and publication trends within the literature have not been systematically evaluated.

Addressing this gap is important to objectively characterize recent shifts in research trends. Therefore, the aim of this bibliometric review was to analyse trends in surgical strategies and technologies—particularly emerging alignment concepts and the transition from navigation to robotic‐assisted TKA—in *KSSTA* from 2015 to 2024, and to assess study designs, levels of evidence (LoE), patient‐reported outcome measures (PROMs) and international collaboration.

## MATERIALS AND METHODS

### Data sources and search strategy

A comprehensive literature search was performed in Scopus on 29 August 2025 to identify English‐language articles published in *KSSTA* between 1 January 2015 and 31 December 2024. The analysis was restricted to *KSSTA*, a leading journal in knee arthroplasty, to ensure a consistent editorial scope for evaluating thematic trends over time. The search terms included ‘total knee arthroplasty’ OR TKA OR ‘total knee replacement’ OR TKR, which were searched in the title, abstract and author keywords fields. The initial search yielded 1074 records. Forty non‐peer‐reviewed materials (editorials, letters and errata) were excluded, leaving 1034 records for eligibility screening, with no duplicates identified using EndNote (Version 21.5; Clarivate).

### Eligibility criteria and data extraction

**Figure 1:**
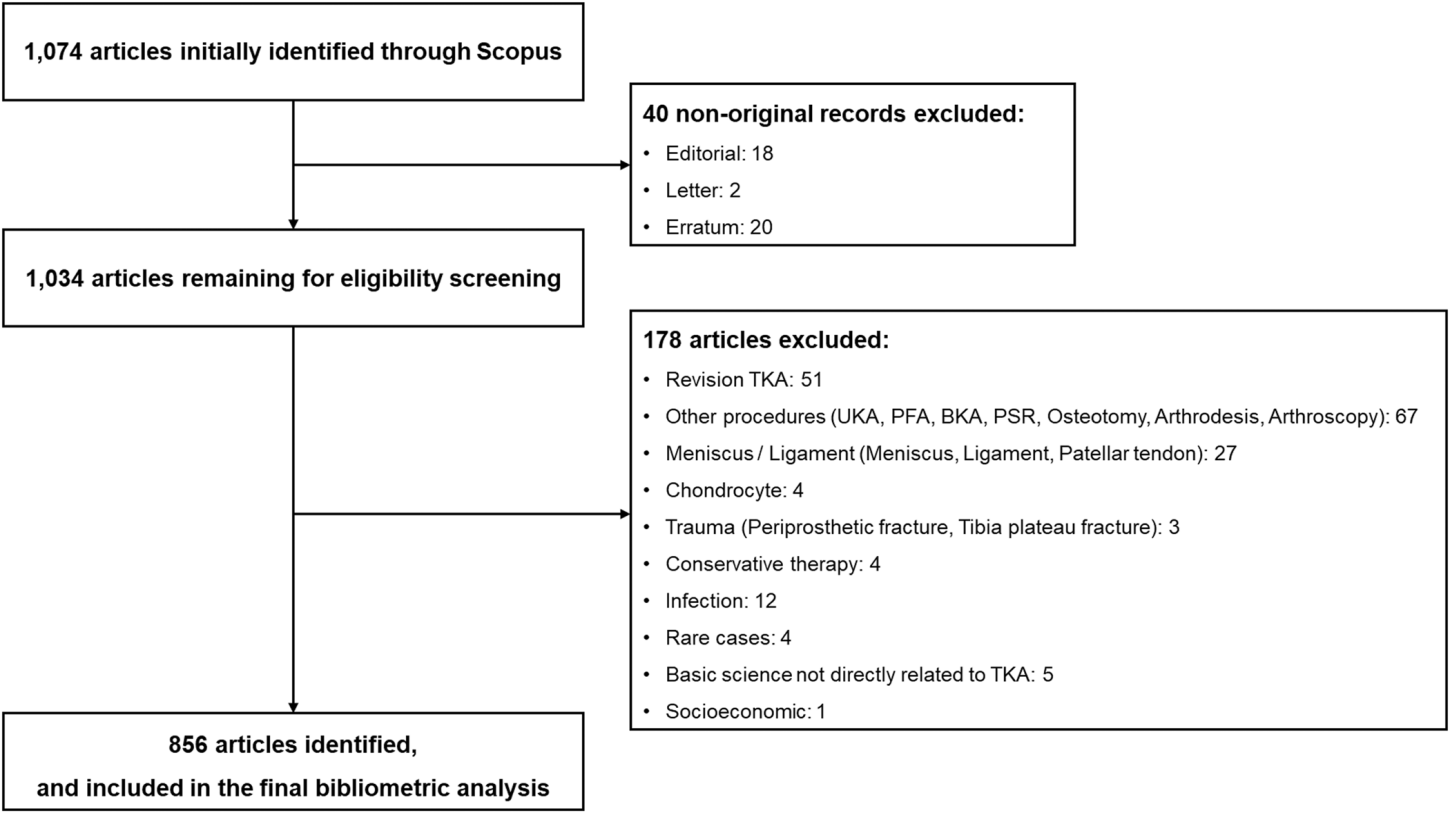
Flow diagram of study selection for TKA articles published in *KSSTA* (2015–2024). BKA, bicompartmental knee arthroplasty; *KSSTA*, *Knee Surgery, Sports Traumatology, Arthroscopy*; PFA, patellofemoral arthroplasty; PSR, patient‐specific resurfacing; TKA, total knee arthroplasty; UKA, unicompartmental knee arthroplasty.

Inclusion criteria were articles published in *KSSTA* between 2015 and 2024 focusing on primary TKA. Studies not mainly addressing primary TKA—such as revision surgeries, other arthroplasty types, other procedures or non‐clinical topics—were excluded, as detailed in Figure 1. After screening, 856 articles were retained for analysis.

**Figure 2:**
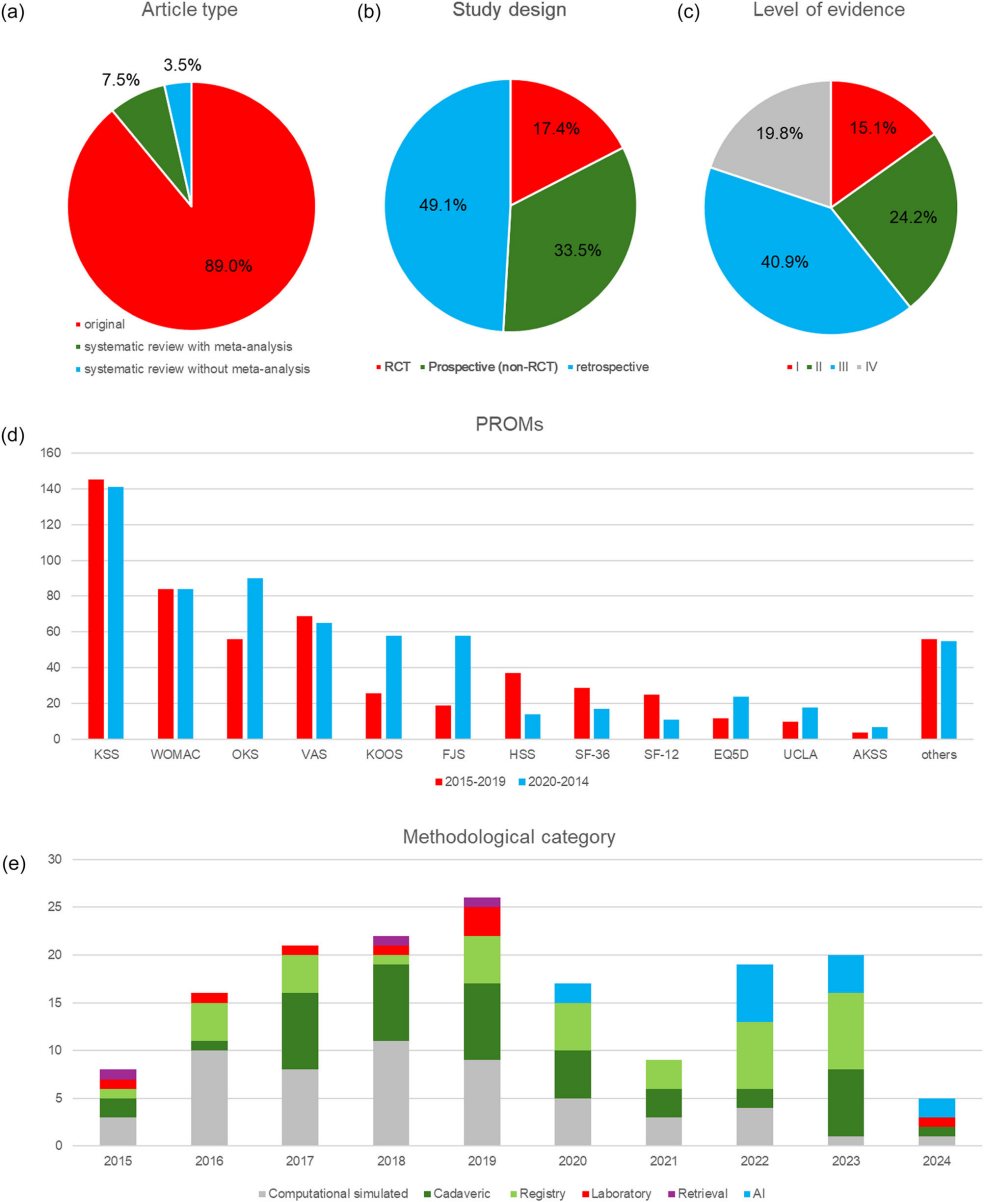
Study characteristics of TKA articles published in *KSSTA* from 2015 to 2024. (a) Article type. (b) Study design. (c) Level of evidence. (d) Distribution of patient‐reported outcome measures (PROMs) in 2015–2019 and 2020–2024. (e) Methodological categories, including computational simulated, cadaveric, laboratory, retrieval and artificial intelligence (AI)‐based studies. AKSS, American Knee Society Score; EQ‐5D, EuroQol 5‐Dimension; FJS, Forgotten Joint Score; HSS, Hospital for Special Surgery Score; KOOS, Knee Injury and Osteoarthritis Outcome Score; KSS, Knee Society Score; *KSSTA*, *Knee Surgery, Sports Traumatology, Arthroscopy*; OKS, Oxford Knee Score; RCT, randomized controlled trial; SF‐36, 36‐Item Short Form Survey; SF‐12, 12‐Item Short Form Survey; TKA, total knee arthroplasty; UCLA, University of California Los Angeles Activity Score; VAS, Visual Analogue Scale; WOMAC, Western Ontario and McMaster Universities Osteoarthritis Index.

For all included records, bibliometric data were automatically extracted from Scopus. Manual classification was also performed for clinical and methodological aspects. Articles were categorized according to alignment concepts (MA, KA or FA) based on the terminology used; if unspecified, they were classified as MA. Surgical assistance technologies were categorized as manual (conventional instrumentation), navigation‐assisted, PSI or robotic‐assisted TKA. Articles involving multiple alignment concepts or surgical technologies were classified by the primary focus; however, if two objects (e.g., KA and FA) were evaluated equally, both were counted. Further classifications included article type (original research or systematic review with/without meta‐analysis), study design (randomized controlled trial [RCT], prospective cohort [non‐RCT] or retrospective cohort) and methodological category (computational simulation, cadaveric study, registry, laboratory, retrieval analysis or AI‐based study). Additionally, the number of knees (original articles), number of included studies (reviews), LoE and PROMs (e.g., Knee Society Score [KSS], Western Ontario and McMaster Universities Osteoarthritis Index [WOMAC], Oxford Knee Score [OKS], Visual Analogue Scale [VAS], Knee Injury and Osteoarthritis Outcome Score [KOOS], Forgotten Joint Score [FJS]; complete list in Figure 2 legend) were recorded.

Two authors (Y. O. and R. E.) independently screened all records for eligibility (*κ* = 0.97) and performed all manual classifications of alignment concepts, surgical assistance technologies and other methodological categories. Disagreements were resolved through discussion and mutual agreement.

### Bibliometric and statistical analysis

For bibliometric mapping and visualization, the dataset was analysed using RStudio (Version 4.5.1; R Foundation for Statistical Computing) with the Bibliometrix package (Version 5.1.1) and VOSviewer (Version 1.6.19; Leiden University). Quantitative analyses included annual publication trends, citation patterns, country‐level productivity and authorship. Network visualizations—including co‐authorship (authors, institutions and countries), keyword co‐occurrence, bibliographic coupling and co‐citation analyses—were constructed using VOSviewer based on association strength normalization.

Descriptive charts were created to illustrate publication volume, study design and topic distribution. Descriptive statistics summarized bibliometric indicators and were presented as counts, proportions, means or medians, as appropriate. Temporal trends in publication volume were evaluated using the compound annual growth rate between the first and last study years. Categorical variables across alignment concepts were compared using the chi‐square test in Easy R (EZR) (Version 1.70; Saitama Medical Center, Jichi Medical University).

## RESULTS

### Publication trends

**Figure 3:**
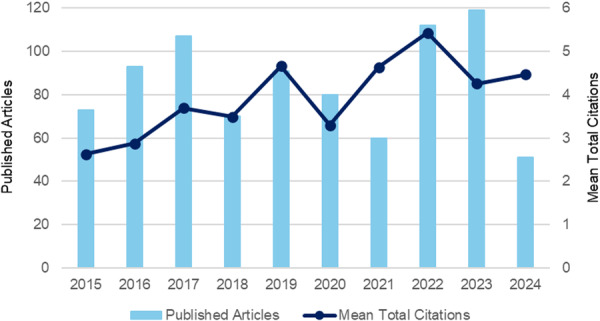
Annual trends in the number of published TKA articles and their mean total citations in *KSSTA* (2015–2024). *KSSTA*, *Knee Surgery, Sports Traumatology, Arthroscopy*; TKA, total knee arthroplasty.

The annual publication volume remained consistently high throughout the decade, peaking in 2023 (119 articles), while the mean citation rate was highest in 2022 (5.43 citations per article) (Figure 3). Although the mean annual growth rate declined slightly (−3.91%), the marked decrease in 2024 likely resulted from incomplete indexing at the time of data extraction rather than from a true decline in research activity [30].

### Geographical distribution

**Figure 4:**
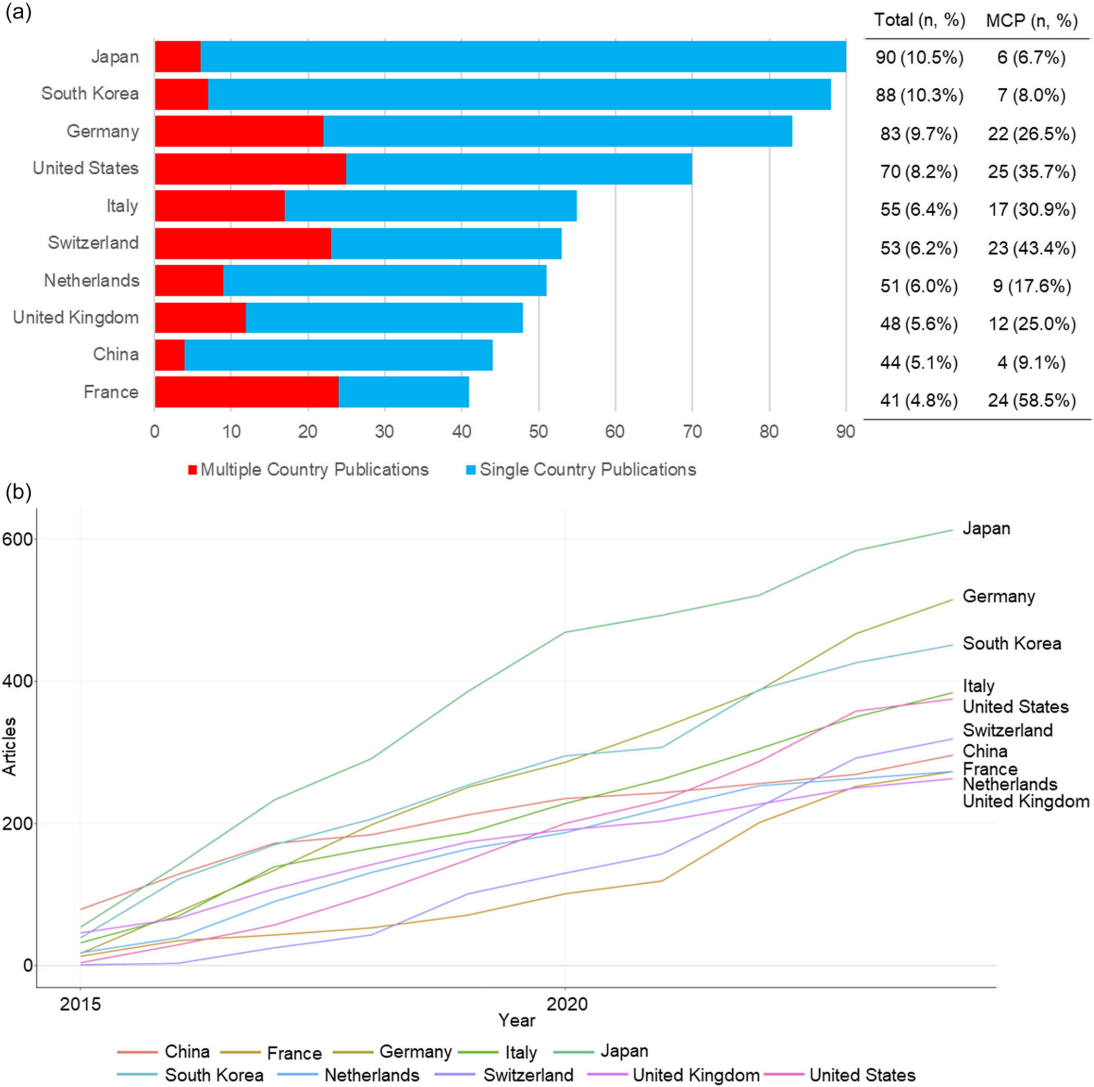
Geographical distribution of authors in TKA articles published in *KSSTA* (2015–2024). (a) Top 10 countries of corresponding authors showing, for each country, the total number of publications and its proportion among all 856 included articles, as well as the number and proportion of MCP. (b) Cumulative publication trends of the top 10 countries based on all contributing authors; multi‐country papers were credited to every participating country. *KSSTA*, *Knee Surgery, Sports Traumatology, Arthroscopy*; MCP, multiple‐country publications; TKA, total knee arthroplasty.

Japan, South Korea and Germany were the most productive countries; the top 10 corresponding‐author countries accounted for 623 of 856 publications (72.8%) (Figure 4a). Asian countries mainly produced single‐country publications, whereas European and North American countries showed comparatively higher rates of multiple‐country publications (MCP). Cumulative publication counts (2015–2024) credited each multi‐country paper to all participating countries (Figure 4b), showing a similar pattern with Japan, Germany and South Korea as the leading contributors.

### Most cited articles

Table 1 lists the most globally cited *KSSTA* articles, including studies addressing functional knee phenotypes [13] (296 citations), robotic‐arm assisted TKA learning curves [16] (239 citations) and projections of future TKA burden on healthcare systems [18] (232 citations). Table 2 presents the most frequently cited references, which predominantly include foundational studies on patient satisfaction [3] (82 citations), constitutional alignment [2] (73 citations) and a radiographic evaluation system [9] (44 citations).

**Table 1:** Top 10 globally cited articles in *KSSTA*.

Rank	Author	Year	Title	DOI	TC	TC per year
1	Hirschmann MT	2019	Functional knee phenotypes: a novel classification for phenotyping the coronal lower limb alignment based on the native alignment in young non‐osteoarthritic patients	10.1007/s00167‐019‐05509‐z	296	42.3
2	Kayani B	2019	Robotic‐arm assisted total knee arthroplasty has a learning curve of seven cases for integration into the surgical workflow but no learning curve effect for accuracy of implant positioning	10.1007/s00167‐018‐5138‐5	239	34.1
3	Klug A	2021	The projected volume of primary and revision total knee arthroplasty will place an immense burden on future health care systems over the next 30 years	10.1007/s00167‐020‐06154‐7	232	46.4
4	Lee WC	2017	The minimal clinically important difference for Knee Society Clinical Rating System after total knee arthroplasty for primary osteoarthritis	10.1007/s00167‐016‐4208‐9	199	22.1
5	Calliess T	2017	PSI kinematic versus non‐PSI mechanical alignment in total knee arthroplasty: a prospective, randomized study	10.1007/s00167‐016‐4136‐8	193	21.4
6	Hirschmann MT	2019	Phenotyping the knee in young non‐osteoarthritic knees shows a wide distribution of femoral and tibial coronal alignment	10.1007/s00167‐019‐05508‐0	184	26.3
7	Liow MHL	2017	Robotic‐assisted total knee arthroplasty may lead to improvement in quality‐of‐life measures: a 2‐year follow‐up of a prospective randomized trial	10.1007/s00167‐016‐4076‐3	160	17.8
8	van der List JP	2016	Current state of computer navigation and robotics in unicompartmental and total knee arthroplasty: a systematic review with meta‐analysis	10.1007/s00167‐016‐4305‐9	157	15.7
9	Zhang J	2022	Robotic‐arm assisted total knee arthroplasty is associated with improved accuracy and patient reported outcomes: a systematic review and meta‐analysis	10.1007/s00167‐021‐06464‐4	153	38.3
10	Lee YS	2017	Kinematic alignment is a possible alternative to mechanical alignment in total knee arthroplasty	10.1007/s00167‐017‐4558‐y	150	16.7

Abbreviations: *KSSTA*, *Knee Surgery, Sports Traumatology, Arthroscopy*; TC, total citations.

**Table 2:** Top 10 locally cited references by *KSSTA* articles.

Rank	Cited references	Citations
1	Bourne RB. Patient satisfaction after total knee arthroplasty: who is satisfied and who is not? *Clin Orthop Relat Res*. 2010;468(1):57–63.	82
2	Bellemans J. The Chitranjan Ranawat award: is neutral mechanical alignment normal for all patients? The concept of constitutional varus. *Clin Orthop Relat Res*. 2012;470(1):45–53.	73
3	Ewald FC. The Knee Society total knee arthroplasty roentgenographic evaluation and scoring system. *Clin Orthop Relat Res*. 1989;(248):9–12.	44
4	Bellamy N. Validation study of WOMAC: a health status instrument for measuring clinically important patient relevant outcomes to antirheumatic drug therapy in patients with osteoarthritis of the hip or knee. *J Rheumatol*. 1988;15(12):1833–1840.	37
5	Berend ME. Tibial component failure mechanisms in total knee arthroplasty. *Clin Orthop Relat Res*. 2004;(428):26–34.	35
5	Berger RA. Malrotation causing patellofemoral complications after total knee arthroplasty. *Clin Orthop Relat Res*. 1998;(356):144–153.	35
7	Almaawi AM. The impact of mechanical and restricted kinematic alignment on knee anatomy in total knee arthroplasty. *J Arthroplasty*. 2017;32(7):2133–2140.	32
7	Behrend H. The ‘forgotten joint’ as the ultimate goal in joint arthroplasty: validation of a new patient‐reported outcome measure. *J Arthroplasty*. 2012;27(3):430–436.e1.	32
7	Dossett HG. A randomised controlled trial of kinematically and mechanically aligned total knee replacements: two‐year clinical results. *Bone Joint J*. 2014;96‐B(7):907–913.	32
7	Hirschmann MT. Functional knee phenotypes: a novel classification for phenotyping the coronal lower limb alignment based on the native alignment in young non‐osteoarthritic patients. *Knee Surg Sports Traumatol Arthrosc*. 2019;27(5):1394–1402.	32

Abbreviation: *KSSTA*, *Knee Surgery, Sports Traumatology, Arthroscopy*.

### Keywords analysis and research trends

**Figure 5:**
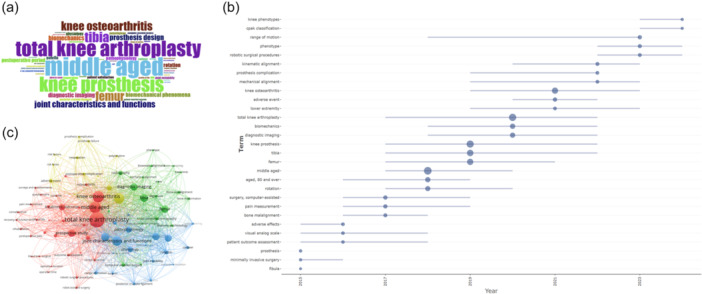
Keyword analysis of TKA articles published in *KSSTA* from 2015 to 2024. (a) Word cloud showing the most frequently used author keywords. (b) Temporal trend analysis of selected keywords over the study period. (c) Co‐occurrence network map of author keywords, illustrating clustered research domains. *KSSTA*, *Knee Surgery, Sports Traumatology, Arthroscopy*; TKA, total knee arthroplasty.

The word cloud (Figure 5a) illustrates the most frequent terms, whereas the temporal trend analysis (Figure 5b) demonstrates changes in keyword prevalence over time. During 2015–2019, keywords such as ‘minimally invasive surgery’ and ‘surgery, computer‐assisted’ (i.e., navigation) were more common, whereas during 2020–2024, terms such as ‘kinematic alignment’, ‘robotic surgical procedures’, ‘phenotype’ and ‘CPAK classification’ increased in frequency.

The co‐occurrence network of author keywords (Figure 5c) highlights the central position of TKA and knee OA and reveals three major research domains: clinical outcomes (e.g., *treatment outcome, quality of life*); biomechanics and imaging (e.g., *femur, tibia, diagnostic imaging*) and surgical innovation (e.g., *computer‐assisted surgery, robotic surgical procedures, kinematic alignment*).

### Alignment concepts

**Figure 6:**
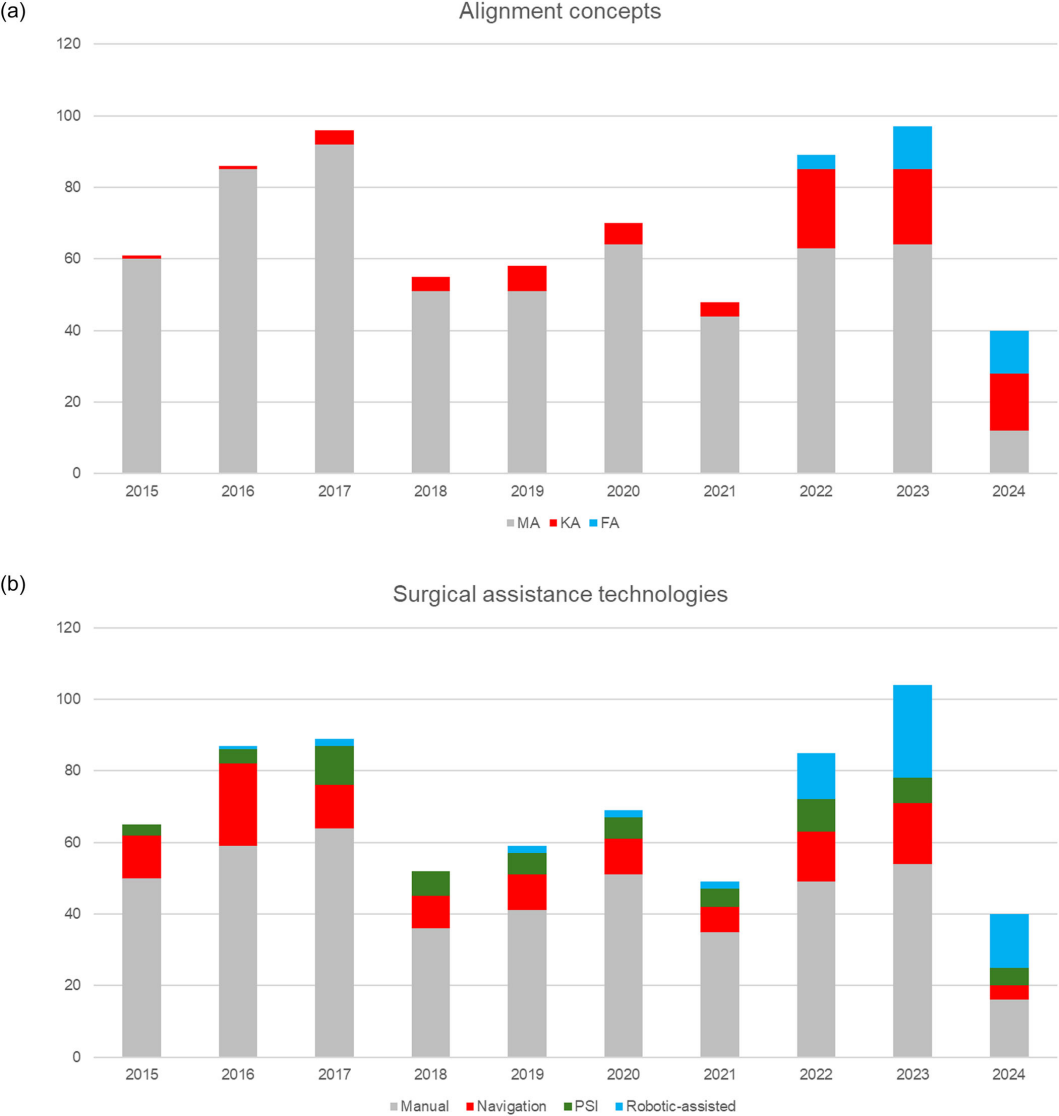
Trends in surgical strategies in TKA articles published in *KSSTA* from 2015 to 2024. (a) Alignment concepts: mechanical alignment (MA), kinematic alignment (KA) and functional alignment (FA). (b) Surgical assistance technologies: manual (conventional instrumentation) TKA, navigation‐assisted TKA, patient‐specific instrumentation (PSI) and robotic‐assisted surgery. *KSSTA*, *Knee Surgery, Sports Traumatology, Arthroscopy*; TKA, total knee arthroplasty.

Figure 6a shows that MA predominated until 2021, whereas KA and FA increased rapidly thereafter. By 2024, KA (16/40 articles, 40%) surpassed MA (12/40 articles, 30%), while FA (12/40 articles, 30%) reached a similar level.

### Surgical assistance technologies

Surgical assistance technologies were reported in 699 of 856 articles (Figure 6b). Manual TKA predominated (455/699, 65%), whereas navigation (118/699, 17%) and PSI (63/699, 9%) maintained smaller yet stable shares. Robotic‐assisted TKA was initially rare but increased rapidly after 2022, totalling 63/699 articles (9%) and peaking at 26/104 articles (25%) in 2023.

### Study characteristics

Original articles dominated (762/856, 89.0%), followed by systematic reviews with meta‐analysis (64, 7.5%) and without meta‐analysis (30, 3.5%) (Figure 2a). Among clinical studies, retrospective studies were the most common (321/654, 49.1%), followed by prospective non‐RCT studies (219/654, 33.5%) and RCTs (114/654, 17.4%) (Figure 2b). Table 3 summarizes sample sizes in original articles and the number of studies included in systematic reviews. LoE were assigned to 766 studies; Level III (313, 40.9%) was the most frequent, followed by Level II (185, 24.2%), Level IV (152, 19.8%) and Level I (116, 15.1%) (Figure 2c). Table 4 shows that MA‐focused studies included a higher proportion of RCTs and Level I evidence, whereas KA‐ and FA‐focused studies were more frequently retrospective and more commonly classified as Level III evidence (*p* < 0.001, = 0.002, respectively).

**Table 3:** Number of cases or included studies.

Category	*n*	Reporting unit	Median [Q1–Q3]	Range (min–max)
Registry studies	38	Cases	15,106 [1692–255,309]	108–2,477,467
AI‐based studies	14	Cases	3433 [1556–8840]	423–334,562
Other clinical studies	609	Cases	111 [64–237]	8–15,807
Systematic reviews with meta‐analysis	64	Studies	14 [9–26.3]	5–129
Systematic reviews without meta‐analysis	30	Studies	11 [8–19]	5–45

*Note*: Number of cases of the knee in original articles and number of included studies in systematic reviews. Data are presented as median values with interquartile ranges (Q1–Q3) and ranges (min–max). Refer to cases or studies. *n* indicates the number of studies in which the information was extractable.

Abbreviation: AI, artificial intelligence.

**Table 4:** Comparison of article characteristics according to alignment concept.

Alignment concepts	MA	KA	FA	*p* value
*n*	586	86	28	
Article type				
Original studies	541 (92%)	82 (95%)	28 (100%)	0.364
Systematic reviews with meta‐analysis	35 (6%)	2 (2%)	0 (0%)	
Systematic reviews without meta‐analysis	10 (2%)	2 (2%)	0 (0%)	
Study design				
RCT	106 (18%)△	5 (6%)^▼^	3 (11%)	<0.001
Prospective (non‐RCT)	177 (30%)	23 (27%)	7 (25%)	
Retrospective	207 (35%)^▼^	36 (42%)	18 (64%)△	
NA	96 (16%)	22 (26%)△	0 (0%)^▼^	
Level of evidence				
I	105 (18%)△	3 (4%)^▼^	1 (4%)	0.002
II	141 (24%)	18 (21%)	6 (21%)	
III	182 (31%)^▼^	38 (44%)△	15 (54%)△	
IV	108 (18%)	16 (19%)	6 (21%)	
NA	50 (9%)	11 (13%)	0 (0%)	

*Note*: Numbers and percentages of articles are presented. *p* values were calculated using the chi‐square test. △ significantly higher proportion; ^▼^ significantly lower proportion (*p* < 0.05).

Abbreviations: FA, functional alignment; KA, kinematic alignment; MA, mechanical alignment; NA, not available; RCT, randomized controlled trial.

PROMs were most frequently assessed using KSS, followed by WOMAC, OKS, VAS and KOOS. The use of FJS rose sharply—from 19 papers in 2015–2019 to 58 papers in 2020–2024—reflecting growing interest in patient‐perceived joint awareness (Figure 2d). Methodological categories showed limited contributions from computational, cadaveric, registry, laboratory and retrieval studies (<7% of all publications). In contrast, AI‐based studies emerged after 2020 and steadily increased, underscoring their increasing representation in contemporary TKA research (Figure 2e).

### Collaboration and network analysis

**Figure 7:**
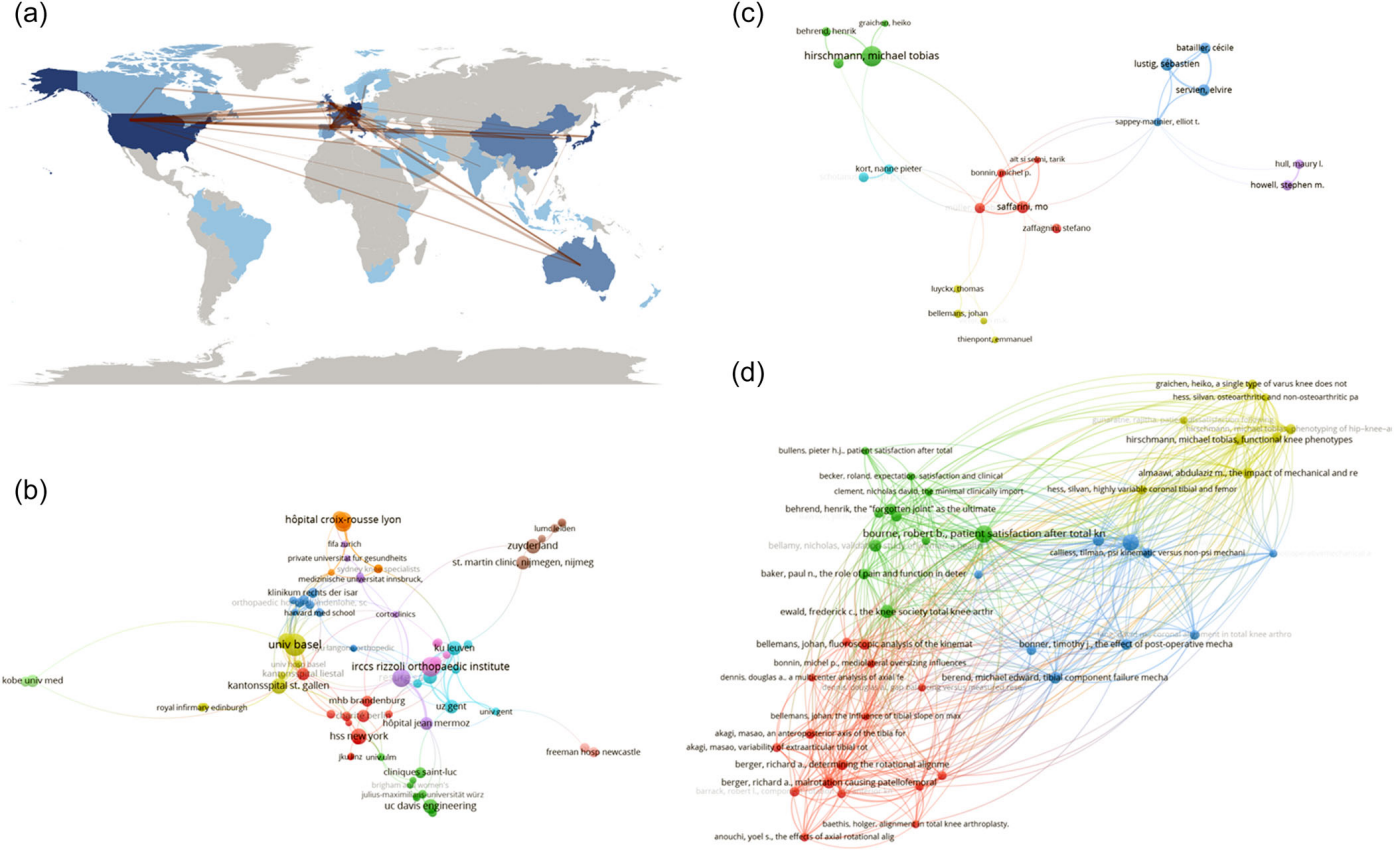
Collaboration and citation networks of TKA articles published in *KSSTA* from 2015 to 2024. (a) International collaboration map showing co‐authorship links between countries. (b) Institutional co‐authorship network, showing major collaborative clusters. (c) Co‐authorship network of leading authors, illustrating connections among leading contributors. (d) Document co‐citation network, highlighting the most influential references in TKA research. *KSSTA*, *Knee Surgery, Sports Traumatology, Arthroscopy*; TKA, total knee arthroplasty.

International collaborations were widely represented in the *KSSTA* literature on primary TKA, with strong partnerships among European countries and between Europe and the United States, alongside additional contributions from Asia and Oceania (Figure 7a).

The institutional co‐authorship network reveals several major clusters led by prominent European centres, indicating frequent inter‐institutional collaboration (Figure 7b). At the author level, the network highlights a limited number of highly connected contributors forming central nodes that link multiple collaborative groups (Figure 7c).

The co‐citation network reveals a limited number of highly cited publications that shaped research directions in this field. Foundational work on patient satisfaction, constitutional alignment and radiographic evaluation was clustered as central references, underscoring its continued influence on contemporary TKA research (Figure 7d).

## DISCUSSION

The most important findings of the present study were three principal trends in primary TKA research within *KSSTA* from 2015 to 2024. First, the alignment philosophy has shifted markedly since 2022, with KA and FA rising from marginal representations to proportions comparable to or exceeding MA by 2024. Second, robotic‐assisted TKA, which was virtually absent before 2021, has expanded rapidly and has become a major research theme. Third, AI‐based studies emerged after 2020 and are steadily gaining recognition, reflecting a broader movement toward data‐driven approaches. These thematic shifts were also evident in the keyword analyses, which increasingly emphasized ‘kinematic alignment’, ‘robotic surgical procedures’, ‘phenotype’, and ‘CPAK classification’, underscoring the growing importance of individualized strategies and technology‐enabled precision.

The marked increases in KA and FA reflect a broader re‐assessment of the long‐standing MA paradigm. MA aims to achieve a neutral mechanical axis, whereas KA seeks to restore the patient's native knee alignment; FA builds on the KA concept while optimizing soft‐tissue balance within predefined safety boundaries. Accumulating evidence has challenged the universality of MA, demonstrating that strict mechanical neutrality does not necessarily ensure higher satisfaction or functional outcomes [1, 35]. Pioneering research on constitutional alignment and knee phenotypes has provided a conceptual foundation for patient‐specific alignment strategies, emphasizing the restoration of the native joint line and soft‐tissue balance [10, 12]. *KSSTA* has increasingly served as a platform for these concepts, underscoring the growing acceptance of individualized alignment philosophies as clinically meaningful alternatives to conventional practice.

The rapid increase of robotic‐assisted TKA after 2021 is one of the most notable developments in arthroplasty research. Early studies focused on learning curves and feasibility [6, 25], whereas more recent studies have emphasized resection accuracy, alignment fidelity and functional outcomes, with short‐term clinical results generally comparable to those of conventional techniques [24, 27]. The global adoption of robotic systems has led to a parallel research stream emphasizing surgical precision and consistency rather than an outright replacement of conventional techniques [7, 11, 23]. This evolution reflects a shift from conceptual debates about alignment targets toward practical questions regarding the achievement of surgical accuracy and consistency across patient populations.

Although AI‐based research is a relatively new field in TKA, it has steadily grown since 2020. In *KSSTA*, these studies employed larger datasets than traditional cohorts and addressed imaging analysis, phenotype classification and outcome prediction [26, 33, 36]. This trend reflects a broader shift toward data‐driven musculoskeletal analytics. The parallel increase in AI and phenotype‐based alignment is notable because computational tools may facilitate large‐scale phenotype identification, guide preoperative planning and support intraoperative decision‐making. While still in its early stages—predominantly retrospective and diagnostic in nature—AI‐based research points toward the future integration of algorithmic support in surgical planning, execution and postoperative monitoring.

Geographic analysis showed that the leading contributors—Japan, South Korea and Germany—remained stable over the decade, which is consistent with an earlier bibliometric review [34]. Interestingly, collaboration styles differed: Asian countries mainly produced single‐country studies, while Europe and the United States showed comparatively higher international collaboration. These patterns may reflect differences in funding, infrastructure and research cultures. Importantly, multi‐country collaborations can enhance the breadth and generalizability of clinical data by including larger and more diverse populations. Although collaboration rates generally remained below 50%, this upward trend highlights the growing recognition of shared expertise and data integration.

Methodologically, most studies were observational (Levels II–III), with relatively few RCTs. Across the study period (2015–2024), the distribution of LoE remained relatively stable, with no clear shift toward higher levels. This likely reflects the practical challenges of conducting large‐scale trials in arthroplasty, where evolving implants and surgical technologies often overtake prospective randomization. Consistent with this pattern, MA‐focused studies included a higher proportion of RCTs and Level I evidence, whereas KA‐ and FA‐focused studies were more often retrospective and classified as Level III evidence. This likely reflects the longer clinical history of MA, while KA and FA remain relatively newer alignment strategies requiring further high‐quality comparative studies. Registry‐ and AI‐based studies, which included much larger sample sizes, provided complementary insights but were limited by data consistency and confounding. Outcome assessment also showed important trends: while traditional scores, such as KSS, WOMAC, OKS, VAS and KOOS, remained common, the FJS gained prominence in recent years. Its growing use aligns with the current emphasis on patient‐centred outcomes, particularly relevant for newer alignment strategies and robotic techniques aimed at achieving a more ‘natural’ joint feel.

This study had several limitations. First, the analysis was restricted to a single journal, *KSSTA*, which ensures internal consistency but may not fully capture global trends across other orthopaedic journals. Second, bibliometric data were extracted solely from Scopus, which was chosen for its compatibility with RStudio and VOSviewer. Although Scopus is recognized as comprehensive in orthopaedics, the use of a single database may introduce indexing and citation biases. Third, bibliometric analyses inherently reflect publication bias, as they rely on published studies and exclude grey literature or negative results. Fourth, the classification of surgical techniques and alignment strategies depended on reporting clarity, potentially leading to misclassification in hybrid or ambiguous cases. Fifth, this study identified themes and publication trends but did not assess the methodological quality or outcomes of the included studies, which were beyond its scope. In addition, most included studies were observational, with relatively few RCTs, which may limit the strength of evidence underlying the observed research trends. Sixth, citation‐based influence is subject to a time lag, potentially underestimating emerging topics, such as FA, robotics and AI; in particular, articles published in 2024 have had limited time to accrue citations, likely leading to lower mean citation counts. Seventh, the apparent decline in 2024 publications likely reflects incomplete indexing at the time of data extraction rather than a true decrease in research activity [30], thereby underestimating the most recent publication volume and growth rate. Finally, although international collaboration was assessed, the corresponding author's country may not accurately represent the distribution of contributions within multi‐institutional studies.

## CONCLUSIONS

In this bibliometric review of 856 TKA articles published in *KSSTA* from 2015 to 2024, research themes have shifted toward personalization and precision. KA and FA increased markedly after 2022, robotic‐assisted TKA rose to nearly one‐quarter of publications by 2023, and AI‐related studies have emerged since 2020. Most studies were observational with Level II–III evidence, indicating that although research themes shifted toward personalization and technology, the overall evidence base remained limited. Continued methodological rigour, standardized reporting and collaborative networks are essential to translate these advances into durable improvements in patient outcomes. Future research should prioritize high‐quality comparative studies to clarify the clinical impact of emerging alignment strategies and technological innovations in TKA.

## AUTHOR CONTRIBUTIONS

Yuma Onoi contributed to the study conception and design, data collection, analysis and drafting of the manuscript. Randa Elsheikh contributed to data collection and revision of the manuscript. Tomoyuki Matsumoto contributed to study design and revision of the manuscript. Ryosuke Kuroda contributed to study design and revision of the manuscript. Rolf Huegli contributed to study design and revision of the manuscript. Andrej Maria Nowakowski contributed to study design and revision of the manuscript. Michael Tobias Hirschmann supervised the study, contributed to study design and revised the manuscript. All authors read and approved the final manuscript.

## FUNDING

The authors have no funding to report.

## CONFLICT OF INTEREST STATEMENT

The authors declare no conflicts of interest.

## ETHICS STATEMENT

This study is a bibliometric analysis of published literature and did not involve human participants, patient data or animal experiments.

## Data Availability

All data analysed during this bibliometric study were derived from publicly available sources. The processed data supporting the findings of this study are included within the published article.
